# Collective foraging of active particles trained by reinforcement learning

**DOI:** 10.1038/s41598-023-44268-3

**Published:** 2023-10-10

**Authors:** Robert C. Löffler, Emanuele Panizon, Clemens Bechinger

**Affiliations:** 1 Fachbereich Physik, Universität Konstanz, 78464 Konstanz, Germany; 2 The Abdus Salam International Centre for Theoretical Physics (ICTP), Strada Costiera 11, 34151 Trieste, Italy; 3 Centre for the Advanced Study of Collective Behaviour, Universität Konstanz, 78464 Konstanz, Germany

**Keywords:** Statistical physics, thermodynamics and nonlinear dynamics, Statistical physics, Colloids, Self-assembly

## Abstract

Collective self-organization of animal groups is a recurring phenomenon in nature which has attracted a lot of attention in natural and social sciences. To understand how collective motion can be achieved without the presence of an external control, social interactions have been considered which regulate the motion and orientation of neighbors relative to each other. Here, we want to understand the motivation and possible reasons behind the emergence of such interaction rules using an experimental model system of light-responsive active colloidal particles (APs). Via reinforcement learning (RL), the motion of particles is optimized regarding their foraging behavior in presence of randomly appearing food sources. Although RL maximizes the rewards of single APs, we observe the emergence of collective behaviors within the particle group. The advantage of such collective strategy in context of foraging is to compensate lack of local information which strongly increases the robustness of the resulting policy. Our results demonstrate that collective behavior may not only result on the optimization of behaviors on the group level but may also arise from maximizing the benefit of individuals. Apart from a better understanding of collective behaviors in natural systems, these results may also be useful in context of the design of autonomous robotic systems.

## Introduction

The self-organization of organisms into functional collective groups is one of the most remarkable examples of how dynamical spatio-temporal patterns can be achieved by only local interaction rules without external control. The abundance of such collective behaviors in many living systems such as birds^1^, fish^2^, insects^3,4^ and bacteria^5^ suggests system-independent overarching organization principles. It has been demonstrated that collective behaviors can be understood in terms of so-called social interaction rules which control local alignment with and attraction towards neighbouring peers^6–8^. Even though such framework is able to reproduce flocking, milling and swarming behaviors, they do not provide an answer why individuals follow such rules. Opposed to a priori motional rules, collective behaviors can be also understood by asking for the specific goals individuals and how they are reached by specific motional behaviors. Examples for such goals are related to foraging^9^, heat preservation^10^ and anti-predation^11–13^. Notably, even when such goals are defined only on the level of individuals, this may lead to advantages for the entire group^13^. The implementation of specific goals into a corresponding framework can be achieved by multi-agent reinforcement learning (MARL), where motional rules of individuals are varied according to a rewarding scheme to an achieve optimal behavior (policy) regarding a given goal^14,15^. This approach has successfully used in computer simulations to investigate e.g. the efficiency of animal flocks^16,17^, cooperative foraging strategies^18^ and predator avoidance^19–21^. In addition to numerical simulations, recently MARL has been also experimentally applied to synthetic systems of active colloidal particles (APs) which mimic many aspects of living systems^22^.

Here, we present an experimental study where we investigate the optimal foraging strategy of 30 APs in presence of a randomly appearing food source. Opposed to previous studies where a priori motional rules have been applied to groups of APs, here we only define a specific task (foraging) which will be accomplished by the group within a MARL framework and without knowing the strategy beforehand. Another advantage of this approach is that system-specific interactions are automatically considered and only little knowledge regarding the details of interactions between APs is required. We demonstrate that the quest of APs to optimize their individual foraging strategy leads to a collective milling motion, similar as observed in living systems^8,23^. Once particles have been trained towards their optimal strategy, a milling behavior is maintained even in absence of food. This suggests the robustness of an optimal strategy regarding variations in the environment.

## Experimental system

In our experiments we have used light-responsive APs which are made from transparent silica particles (diameter $\sigma =6.3$ μm) being coated on one side with a 80 nm light absorbing carbon cap. They are suspended in a water-lutidine mixture contained in a thin sample cell whose temperature is kept below the mixture’s lower demixing point $T_c \approx 34$ °C. Due to gravity, the APs settle to the bottom plate of the sample cell where they perform a two-dimensional motion. When illuminated with a focused laser beam, the caps are selectively heated above $T_c$ which results in a temperature gradient which leads to local demixing and eventually self-propulsion of APs^24^. By scanning the laser beam across all particles and individually adjusting its intensity and position relative to the carbon cap, the magnitude and the direction of the propulsion velocity of APs can be controlled independently (Methods).

**Figure 1:**
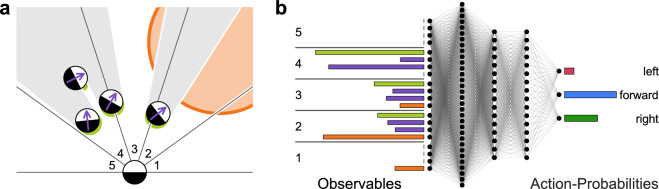
Multi-agent reinforcement learning. (**a**) APs retrieve information about their local environment by visual perception. A 180 °C vision cone is divided into five segments for each of which particles observe the density of neighboring particles (lime green), their mean orientation (purple) and the food source (orange). The observation strength is determined by the sum of imaged fractions of objects weighted with inverse distance (Methods). Neighboring particles also obstruct vision towards objects further away (gray areas). (**b**) The resulting set of 20 observables for neighbor presence, neighbor orientation (as two-element vector) and food serves as input to the neural network (policy), which is modeled as a dense ANN with three hidden layers. The output of the policy represents the probability distribution for an appropriate action, being either left turn (red), forward motion (blue) or right turn (green). The figure was created using MatLab (version 2022B), https://www.mathworks.com/products/matlab.html.

Because the positions and orientations of all particles are continuously determined via real-time tracking, the local configuration of all particles are permanently recorded. This information can be allocated to each AP to gain knowledge regarding its environment. To quantify the position and orientation of peers and the location of the food source, each AP virtually senses its environment by a vision cone which covers $180$° (aligned with the AP orientation) which is divided in five equal sections (Fig. 1a). For each section, the particle determines the density and mean orientation of neighbors and the imaged fraction of the food source which has a diameter of 80 μm (Fig. 1a, orange region). To yield a realistic visual perception, we have chosen a metric perception model where objects in the environment contribute with their inverse distance. Such signal decay is motivated by its established role in the swarming of insects^25^. To make this model even more realistic, we have considered visual obstruction effects due to the finite size of the APs (Fig. 1a, gray areas; Methods).

At each instance of time, every AP (agent) chooses one of three possible motional actions, depending on the instantaneous visual cues described above. These actions are: (i) move straight forward, (ii) turn left and (iii) turn right respectively. Even though such action space appears to be rather simple, it resembles the discrete motional behavior of several bacteria^26^. During the turning motions, APs also exhibit a forward motion which results in a radius of curvature of about 10 μm. The choice of a specific action is determined via an artificial neural network (ANN) which delivers the “policy”, i.e. the probabilities for selecting one of the above actions (Fig. 1b). This ANN, called “actor”, is optimized by the framework of clipped proximal policy optimization (PPO, Methods) to maximize the sum of future rewards of each agent, known as return, which is the primary optimization objective in reinforcement learning. In our study, the return $G = \sum _t \gamma ^t R_{t+1}$ is discounted by $\gamma = 0.97$. To optimize the policy towards a given task (goal), one hast to define the instantaneous reward. In our specific example of foraging the reward $R_t$ is defined to be positive when the AP’s center of mass is within the circular area of the food source (for a precise reward definition we refer to the Methods). To reproduce the consumption of food, the capacity of the food source decreases depending on the time and number of APs within this region. When the food source is exhausted, another food source appears at a new randomly chosen location within the experimental field of view (Methods).

## Results

**Figure 2:**
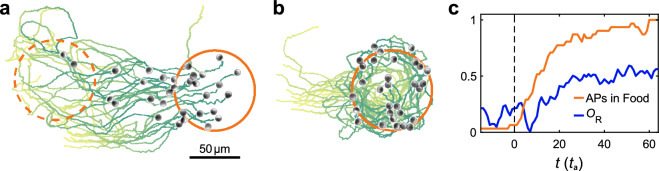
Collective behavior. (**a**, **b**) Experimental snapshots of trained APs, moving as a flock form one food source to the next and forming a milling state within the food source. Snapshots show microscope images of APs, annotated with trajectories during the last 40 actions (colored from bright to dark evolving in time) and previous (dashed orange) and current (solid orange) location of the food source (see also supplementary video S1). (**c**) Strength of milling characterized by order parameter $O_\text {R}$ (blue line, see main text), which strongly increases with the fraction of APs located inside the food source (orange line) once the flock has arrived at the food source (dashed vertical line). The figure was created using MatLab (version 2022B), https://www.mathworks.com/products/matlab.html.

In general, each experiment starts with training sequences, where the ANN is initialized with random weights which leads to random actions of the APs. Over time, agents learn which actions are leading to an increased return depending on the observables. Such training is conducted until the weights within the ANN converge to an optimized policy. In the case of our experiments, such training takes about 60 h of measurement time. Figure 2a shows an example of the APs trajectories resulting from a learned policy when moving from a depleted food source on the left (dashed circle) to another one appearing on the right (solid circle). The brightness of the trajectories (yellow green to dark green) indicates the evolution in time. As expected from the APs greedy strategy, they follow an almost direct path from the depleted to the new food source which results in high relative orientational alignment. Such behavior will be called a flocking state in the following. Once particles have arrived at the food source, their motion changes into a milling motion, i.e. rather circular trajectories within the food area (Fig. 2b, see also Suppl. Video S1). The group’s milling motion can be quantified by a rotational order parameter

1 $$\begin{aligned} O_\text {R} = \frac{1}{N} \sum _i ({\hat{r}}_i \times {\hat{u}}_i ) \cdot {\hat{e}}_z \end{aligned}$$

where *N* is the number of particles, ${\hat{r}}_i$ is a unit vector pointing from the group center to the *i*-th AP, ${\hat{u}}_i$ is the unit vector denoting the APs orientation and ${\hat{e}}_z$ is the unit vector perpendicular to the sample plane. Figure 2c shows the temporal evolution $O_\text {R}$ together with the number of APs within the food source. In our experiments we observe that milling is maintained until the food becomes entirely depleted.

**Figure 3:**
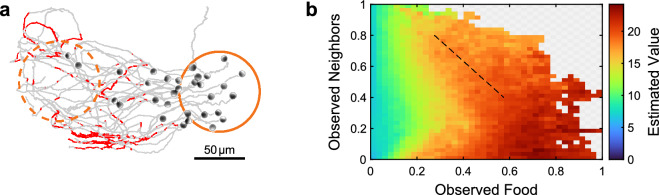
Vision obstruction and value function. (**a**) Experimentally measured trajectories (grey) of a group of trained APs which are moving towards a food source (orange circle). Red parts of the trajectories indicate instances where the vision of the corresponding AP towards the food source is blocked by peers. (**b**) Estimated expected return (value of state) in dependence of sum over neighbor density observables and sum of food observables, respectively. Although reward is only correlated to food, expected return is also correlated to neighboring particles (indicated by dashed line). All data is sampled from experimentally measured configurations, gray-checkered background marks combinations of observables which did not occur in experiments. The figure was created using MatLab (version 2022B), https://www.mathworks.com/products/matlab.html.

At first glance, the observation of a collective flocking and milling behavior is surprising because the reward (and the maximized return) is defined and optimized only on the level of single APs. To rationalize the flocking behavior, one has to consider that not all particles have a full, i.e. unobstructed view towards the food source due to particle sight-blocking by peers. To compensate for such lacking information, APs with zero (or limited) food perception can increase their chance of steering towards the food source by following their peers. This sounds a reasonable strategy because peers may have a better, i.e., more direct view towards the food. This idea is supported by Fig. 3a which shows the trajectories of APs moving towards a food source. The trajectories are labeled in red for those APs whose vision towards the food source is blocked by their peers. This applies to up to $10\%$ of the APs in the tail of the group. As mentioned above, this lacking information is then compensated by following their peers which then leads to alignment, i.e. a flocking state.

The above arguments are quantitatively supported by the so-called value function (an auxiliary ANN which is part of the RL framework (Methods)) which provides an estimate for the return depending on a specific configuration. Figure 3b shows the measured estimated value obtained from trajectories as a function of the observables characterizing the amount of neighbors and food perceived by an individual. Obviously, this quantity is high for large perception of the food since under such conditions APs are getting closer to the rewarded food source. In addition, a proximity to peers also leads to an increase of the estimated value, even when the perception of the food source is low (see arrow). This must stem from instances where the APs are close to food but their vision is mostly blocked by other peers. APs then learn to align and follow peers—leading to flocking—driven by the realization the large perception of peers leads to higher gains.

After having explained why a flocking behavior is part of an ideal food-searching strategy, we now discuss the organization of the APs after they have reached the food source. Because the reward requires the permanently moving APs to be localized within the circular food source, this naturally leads to a milling motion of the group, in agreement with our experiments. Note, that a single sense of rotation is randomly selected during the training process and maintained afterwards.

**Figure 4:**
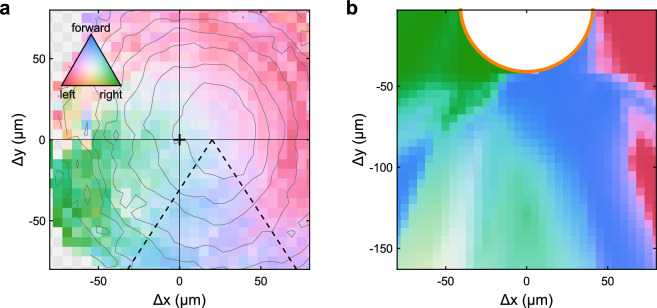
Policy map. Probabilities for an individual AP to choose one of the three actions (forward, left turn, right turn) in respect to its current configuration. (**a**) Action probabilities for a north-facing AP depending on its position relative to the center of mass of the particle group. Data is sampled from experimental trajectories, the gray contour lines indicate the number of occurrences of the respective configuration with most particles being found counter-clockwise aligned, i.e. to the right of the group center. The dashed lines serve as a guide to the eye. (**b**) Action probabilities for a single particle without perception of peers approaching a food source. The figure was created using MatLab (version 2022B), https://www.mathworks.com/products/matlab.html.

The milling behavior is also reflected in the spatial distribution of actions performed by the particles. Figure 4 shows the measured actions of APs (within their frame of reference) for a counter-clockwise group rotations within the food source. In fact, the steering direction of the APs is slightly offset from the group center which is important to create a tangential motional component. Such behavior is in excellent agreement with previously observed milling based on social interaction rules^27,28^. Interestingly, formation of milling motion is already initiated from the beginning when individuals enter the food source. This is shown in Fig. 4b which shows the spatially resolved actions of a single particle (i.e. without perception of peers) near the food source obtained from the optimized policy. Such conditions typically apply to the leading particles of the flock approach the food. As seen by the blue region (forward motion), the AP only moves straight towards the food source when located to the right hand side of the food source. Otherwise APs reorient accordingly, to enter the food always from the right. Thus behavior is ideal for the development of counter-clockwise milling.

**Figure 5:**
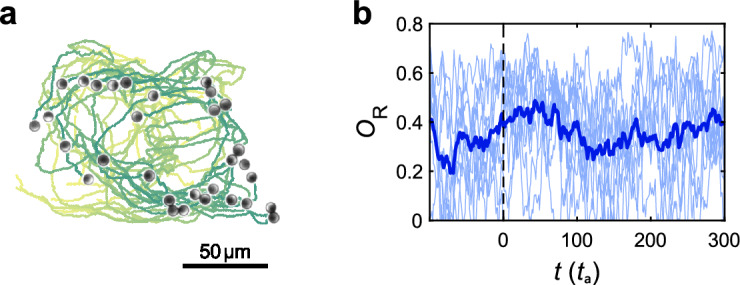
Behavior in the absence of food. (**a**) APs freely milling in the absence of food. The Snapshot shows a microscope image of APs, annotated with trajectories during the last 40 actions (colored from bright to dark evolving in time, see also Suppl. Video S2). (**b**) Orientational order parameter $O_\text {R}$ for several time series (thin blue lines) and their average (blue line) for a scenario where particles mill inside a food source for $t < 0$, but also keep a stable milling formation after the food source is removed ($t>0$).

As shown above, the optimal policy of the above discussed food-searching problem leads to a milling behavior within the food source. Notably, the identical policy leads to a milling behavior even in absence of food. Figure 5a shows the resulting trajectories of a group of APs which has been released from a hexagonal initial positional configuration with random orientation. Even though the food perception of all APs is set to zero, the particles show a very similar milling as reported in Fig. 2b (see also Suppl. Video S2). The corresponding rotational order parameter can reach values up to 0.6 which is similar to the value within a food source (Fig. 5b). In contrast to Fig. 2b, here the particle density is reduced near the center of rotation. This behavior can be rationalized by considering the above mentioned orientation alignment of APs with no view towards the food which is enforced by the policy. In absence of food, this results in a ’circular’ flock where each particles follows its peers in front.

## Discussion

In our experiments we have investigated the ideal strategy of active particles to localize a randomly appearing finite food source using MARL. After training is complete, the optimized policy leads APs to move towards the food source in a aligned flock. Once the food source is reached, a milling motion develops within the circular area of the food. Such milling motion appears to be rather robust and also occurs in absence of the food source. Often collective states are described as a result of social interaction rules where particles aim to adjust their motion to that of peers. In our work, we demonstrate that similar states can also result from a mere selfish motivation which in our case is the individual (not that of the group) food uptake which is rewarded in our policy. The main reason why collective states may arise even under selfish conditions is, that individuals benefit from considering the motion of their peers. In particular when relevant information to reach a desired goal is missing (e.g. by visual obstruction of neighbors), this can be partially compensated by adjusting the behavior to that of peers which may have more (or different) information regarding their surrounding. The fact, that the above behavior is observed in an experimental system demonstrates its robustness regarding thermal noise, hydrodynamic and steric interactions but also unavoidable variations in the properties of the APs. Such deviations from an ideal and monodisperse behavior which is typically not included in numerical simulations is certainly of importance in living but also artificial robotic collectives comprised of hundreds or even thousands of group members. In our case, phoretic interactions of APs being in immediate contact is known to deteriorate their ability to rapidly change their steering direction. Although close particle distances have not been penalized within our reward definition, after the training has been completed, the optimized policy strongly avoided particle collisions which is important to enhance the ability of individuals to suddenly change their motion in response to a newly appearing food source. As a possible extension of our study, one could consider food sources which are distinguished by their physical properties. One possibility could be to rely on physical, impenetrable objects, such as additional disks, which then would be “virtually depleted” (in post-processing) as the particles enter in contact with them. This, however, would require major experimental modifications beyond the scope of the current work.

## Methods

### Active particles

Light activated active particles are fabricated from commercially obtained 6.2 μm silica spheres which are capped with a layer of 80 nm Carbon on one hemisphere. They are then suspended in a thin sample cell in a critical binary mixture of water and lutidine which is kept close to its lower demixing point at 34 °C. Upon illumination of the particles, the capped hemisphere is heated above the critical point, leading to local demixing of the fluid and, thus, self-propulsion of the particle^24,29^. To enable individual steering of particles, a feedback-loop is used: Images of the sample are taken at a rate of 5Hz; live image-analysis and particle tracking are performed to provide particle trajectories to the reinforcement-learning algorithm; finally an acousto-optic deflector, scanning the particles at a rate of 10MHz is used to illuminate individual particles with a slightly defocused 532 nm laser beam. The beam waist in the particle plain is about 4 μm. In order to apply active steering to the particles, the laser spot is either offset to the capped side of the particles for a stabilized forward motion, or two laser spots per particle with different intensities to either side are used to generate a heat gradient within the carbon cap and therefore anisotropic demixing, resulting in an active torque^27,30^. Note that due to the weak intensities and the defocused beam used in the experiment, no optical forces are applied to the particles. Throughout the measurements we keep the number of particles constant to $N=30$. To ensure that no particles get lost and no new particles diffuse into the measurement area, effective boundary conditions are applied by means of the feedback loop: Particles entering the field of view get propelled back out, while particles reaching the boundary of the measurement area from inside get rotated and propelled back in, creating effective reflective boundary conditions^30^. Note that these actions overrule the RL policy and consequently, the trajectories of particles reorienting due to the boundary condition are not included when training the policy. Before the start of each measurement, particles are propelled to homogeneously spaced starting positions at the center of the measurement area.

### Reward and observables definition

The virtual food source used for the reward definition has a fixed diameter of 80 μm. The capacity is set to 1000 rewards, which equals to an average depletion time of about 15 min. Upon depletion, the food source is relocated to a new random location within the available experimental accessible space of about 300 μm by 400 μm, with a minimum distance to the boundary and at least 120 μm away from the last location. Particles at position $\vec {r}_i$ are rewarded strictly if their center is within the food source,

2 $$\begin{aligned} R_{i,t} = {\left\{ \begin{array}{ll} 1 &{} (|\vec {r}_i-\vec {r}_\text {food}| <= 80\,\upmu \!\text {m}) \\ 0 &{} \text {otherwise} \end{array}\right. } \,. \end{aligned}$$

The observables $\vec {O}_{i,t}$ which represent the visual input of the agent *i* at time step *t* are calculated per section *m* of the vision cone in respect to particle orientation $\theta _i$, as

3 $$\begin{aligned} \vec {O}_{i,t}&= (\vec {f}_{i,t}, \vec {p}_{i,t}^\text {d}, \vec {p}_{i,t}^\text {sin}, \vec {p}_{i,t}^\text {cos}) \,,\end{aligned}$$

4 $$\begin{aligned} f_{i,t,m}&= \min \left( \frac{\sigma _\text {food}}{|\vec {r}_i - \vec {r}_\text {food}|}\,, 1\right) g_{i,\text {food},t,m}\,,\end{aligned}$$

5 $$\begin{aligned} p^\text {d}_{i,t,m}&= \sum _j \frac{\sigma _\text {part.}}{|\vec {r}_i - \vec {r}_j|}\, g_{i,j,t,m} \,, \end{aligned}$$

6 $$\begin{aligned} p^\text {sin}_{i,t,m}&= \sum _j \sin (\theta _i-\theta _j) \frac{\sigma _\text {part.}}{|\vec {r}_i - \vec {r}_j|}\, g_{i,j,t,m}\,, \end{aligned}$$

7 $$\begin{aligned} p^\text {cos}_{i,t,m}&= \sum _j \cos (\theta _i-\theta _j) \frac{\sigma _\text {part.}}{|\vec {r}_i - \vec {r}_j|}\, g_{i,j,t,m} \,, \end{aligned}$$

where $\sigma$ is the food/particle diameter and $g_{i,j,t,m}$ denotes the relative amount of particle *j* being visible within section *m* of particle *i*’s vision cone (see Fig. 1a). Note, that $\vec {p}_i$ can not diverge, as particles cannot get closer than $\sigma$.

### The multi agent reinforcement learning (MARL) algorithm

A natural theoretical framework to study how collective motion emerges from the solution of tasks is that of Multi Agent Reinforcement Learning. While a comprehensive overview of it is outside the scope of this work (see^15^ for a recent review), here we provide the basic information relevant for our study. In our setup agents perform actions in response to environmental cues, the observables $\vec {O}_{i,t}$ defined above. Actions are consequently drawn from the policies $a_{i,t} \sim \pi _i(a {{\,\mathrm{|}\,}}\vec {o}_{i,t})$, which encode the behaviors of the agents. Here we employ the centralized-training, decentralized-execution paradigm^14^, where policies are shared, i.e. $\pi _i = \pi _j = \pi$. While the policy is shared, agents do not communicate and are fully independent in the choice of action, which depends only on the individual local environment (through the observables). The agent’s actions therefore can be different at any time.

Through experience, the agents optimize their policies to a specific goal objective, defined through the reward functions. The past experience is stored in the “trajectories”, which are the temporal series of observations, actions and rewards obtained by each agent during a whole episode. The process of optimization is done through stochastic policy-gradient algorithm: The agents’ policies are learned using the Proximal Policy Optimization (PPO) actor-critic learning algorithm^31^, together with the generalized advantage estimation (GAE)^32^. The two artificial neural networks (ANNs) corresponding to the “actor” (i.e. the policy $\pi$) and “critic”, share the same input and hidden layers, with the input having 20 nodes corresponding to the observable vector $\vec {o}_i$ (see “Reward and observables definition”). The hidden layers are dense layers with 32, 16 and 16 nodes respectively and a ReLu non-linear activation function. The output layer of the critic is a scalar, while the actor ANN has a final layer of the size of the number of actions, with a softmax activation. Both ANNs are initialized with random weights. The parameter for the reward discount is set to $\gamma =0.97$, and that for the estimator of the GAE as $\lambda =0.97$. For a more detailed description of the code and algorithm used, see the Methods in^13^. While here we use an experimental realizations of the system to obtain trajectories of data, the algorithmic part is effectively equivalent.

The global state of the system at any given time is captured by the imaging and tracking algorithm in the feedback loop. The difference between states of consecutive time steps is given by the physical evolution of the experimental system. As the RL algorithm requires a significant difference between consecutive states in order to adjust the policy in a meaningful way, actions are drawn at a lower frequency than the steering feedback loop. Namely, new actions $a_{i,t}$ are assigned to all particles every 10 seconds based on their local observables $\vec {o}_{i}(s_t)$ and kept constant until the next update. This corresponds to a forward motion of approximately $1\sigma$ per action and a possible rotation of approximately 22 °C per action. If a particle reaches the boundary of the measurement area, it is considered lost to the RL framework and its trajectory is ended. After it is properly reoriented and swimming back into the measurement area, a new trajectory is started, such that the RL algorithm can not exploit the boundary condition for its strategy. Note that, while this situation is common in early training, due to the general attraction towards the food source, events of particles reaching the defined boundary area become less likely towards the end of policy optimization. Every 120 time steps (corresponding to 20 minutes of experimental time) all recorded trajectories are evaluated and the gathered experience is used to improve the policy.

It is important to note that the RL algorithm optimizes the policy in respect to a single particle to maximize reward gained over time. This particularly means that in some circumstances an action might be preferred which does not maximize the reward in the immediate next step, but only over the long term. In respect to the given task, it is not only important for a particle to stay inside the current food source, but also to be able to reach the next food location as fast as possible when the current one is depleted.

### Supplementary Information

Supplementary Information 1.

Supplementary Information 2.

Supplementary Information 3.

## Data Availability

The datasets generated and analysed during the current study are available from the corresponding author on reasonable request.
