# Supplementary material for: Collective foraging of active particles trained by reinforcement learning
Source: Sci Rep. 2023 Oct 10;13:17055. doi: 10.1038/s41598-023-44268-3 (PMC10564893; doi:10.1038/s41598-023-44268-3)
Supplement: Supplementary file 1 — Supplementary Information 1. [file 41598_2023_44268_MOESM1_ESM.pdf]

Description of Supplementary Videos

Supplementary Video S1. Final Policy. Microscope recording of APs behaving according to the final policy after training has been completed. APs are annotated with trajectories during the last 40 actions (colored from bright to dark evolving in time) and current location of the food source (orange circle). The recording is speed up to 240x real time, scale bar is 20µm. The movie was created using MatLab (version 2022B), https://www.mathworks.com/products/matlab.html.

Supplementary Video S2. Behavior in Absence of Food. Microscope recording of APs behaving according to the final policy if no food source is present in the experimental environment. APs are annotated with trajectories during the last 40 actions (colored from bright to dark evolving in time). The recording is speed up to 240x real time, scale bar is 20µm. The movie was created using MatLab (version 2022B), https://www.mathworks.com/products/matlab.html.
